# Biological and therapeutic insights from animal modeling of fusion-driven pediatric soft tissue sarcomas

**DOI:** 10.1242/dmm.050704

**Published:** 2024-06-25

**Authors:** Jack P. Kucinski, Delia Calderon, Genevieve C. Kendall

**Affiliations:** ^1^ Center for Childhood Cancer Research, The Abigail Wexner Research Institute, Nationwide Children's Hospital, Columbus, OH 43215, USA; ^2^ Molecular, Cellular, and Developmental Biology PhD Program, The Ohio State University, Columbus, OH 43210, USA; ^3^ Department of Pediatrics, The Ohio State University College of Medicine, Columbus, OH 43215, USA

**Keywords:** Fusion Oncogene, Pediatric oncology, Sarcoma, Transgenic animal models

## Abstract

Survival for children with cancer has primarily improved over the past decades due to refinements in surgery, radiation and chemotherapy. Although these general therapies are sometimes curative, the cancer often recurs, resulting in poor outcomes for patients. Fusion-driven pediatric soft tissue sarcomas are genetically defined by chromosomal translocations that create a chimeric oncogene. This distinctive, almost ‘monogenic’, genetic feature supports the generation of animal models to study the respective diseases *in vivo*. This Review focuses on a subset of fusion-driven pediatric soft tissue sarcomas that have transgenic animal tumor models, which includes fusion-positive and infantile rhabdomyosarcoma, synovial sarcoma, undifferentiated small round cell sarcoma, alveolar soft part sarcoma and clear cell sarcoma. Studies using the animal models of these sarcomas have highlighted that pediatric cancers require a specific cellular state or developmental stage to drive tumorigenesis, as the fusion oncogenes cause different outcomes depending on their lineage and timing of expression. Therefore, understanding these context-specific activities could identify targetable activities and mechanisms critical for tumorigenesis. Broadly, these cancers show dependencies on chromatin regulators to support oncogenic gene expression and co-opting of developmental pathways. Comparative analyses across lineages and tumor models will further provide biological and therapeutic insights to improve outcomes for these children.

## Teaser

**Summary:** Fusion-driven pediatric soft tissue sarcomas are aggressive and lack targeted therapies. Transgenic fusion-driven animal tumor models are a platform to understand tumorigenic mechanisms and translate findings into therapeutic strategies.

## Introduction

Pediatric cancers are a devastating diagnosis and result in an unacceptable annual loss of 10-20 million years of expected life globally (Alvarez et al., 2022; Force et al., 2019). Overall outcomes for children with cancer have improved through the decades, largely due to refinements in surgery, radiation and first-line chemotherapeutic agents (Hudson et al., 2014). However, pediatric cancer survivors face adverse long-term health effects and an increased risk of secondary cancer formation due to treatment toxicity (Hudson et al., 2014; Suh et al., 2020). Therefore, there is a critical unmet need for targeted therapies to improve outcomes for these children and minimize negative off-target effects and lifelong consequences. Pediatric cancers often have a low mutational burden compared to that of adult tumors, and tumor histology reflects features of abnormal development (Thatikonda et al., 2023; Gröbner et al., 2018; Filbin and Monje, 2019). However, each disease has a slightly different genetic driver and disease presentation; given the rarity of subtypes, it is challenging to design and test drugs specific to each cancer (Laetsch et al., 2021). Thus, a deeper understanding of disease biology is required to improve patient outcomes and therapeutic recommendations.

**Fig. 1.**
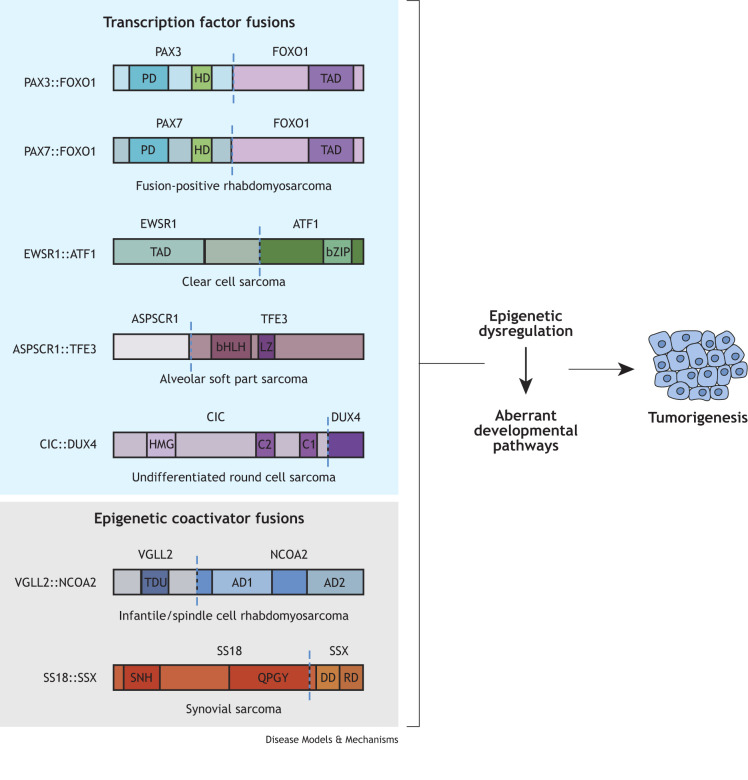
**Common pediatric soft tissue sarcoma fusion oncoproteins.** Fusion oncoproteins are categorized by the known or hypothesized activity of the fusion partner. The blue vertical dashed line represents the breakpoint in each fusion protein and each color denotes the relevant protein domains for each fusion partner. Differentially colored regions represent a known domain in each fusion oncoprotein. Fusion oncoproteins are not drawn to scale to each other. Abbreviations: AD1/AD2, transcriptional activation domains; bHLH, basic helix-loop-helix DNA-binding domain; bZIP, basic leucine zipper DNA-binding domain; C1/C2, capicua homology domains; DD, divergent domain; HD, homeobox DNA-binding domain; HMG, high-mobility group domain; LZ, leucine zipper domain; PD, paired-box DNA-binding domain; QPGY, glutamine, proline, glycine and tyrosine domain; RD, repression domain; SNH, N-terminal homology domain; TAD, transactivation domain; TDU, tondu domain.

Soft tissue sarcomas are rare in children and occur in connective tissues such as muscles, fat, blood vessels, skin and tendons (Loeb et al., 2008). One common genetic driver is a chromosomal translocation mutation that inappropriately joins two genes and generates a chimeric protein, or fusion oncoprotein, with neomorphic functions that have transformative capabilities (Mitelman et al., 2007). These fusion oncogenes are present in 20-49% of sarcomas and are often used for tumor diagnosis and as prognostic indicators (Watson et al., 2018; Mitelman et al., 2007; Vellichirammal et al., 2021). These defining fusion oncogenes provide an opportunity to model rare diseases by introducing the respective oncogene into animal systems to understand the mechanisms of tumorigenesis. From these animal models, it has become clear that the oncogenic potential of these molecular drivers varies between cellular lineages and developmental timing, suggesting that tumorigenesis requires a discrete cellular context (see Box 1) (Amatruda, 2021). This Review will focus on fusion oncogenes that contain transcription factors or chromatin regulators and have genetic animal models (Fig. 1). The discussed cancers include rhabdomyosarcoma (RMS; fusion-positive and infantile), synovial sarcoma (SS), undifferentiated small round cell sarcoma (URCS), alveolar soft part sarcoma (ASPS) and clear cell sarcoma (CCS). For a summary of tumor models, see Table 1.

**Box 1. Lineage-specific susceptibilities to tumorigenesis.**

Pediatric cancers have characteristics of developmental precursor cells, and understanding their cell(s) of origin would help identify oncogenic dependencies (Amatruda, 2021). Animal tumor models have shown that cellular lineages have varied responses to fusion oncogene expression. With the zebrafish fusion-positive rhabdomyosarcoma (FP-RMS) model, fusion oncogene expression resulted in a range of tumor types depending on its promoter, with FP-RMS only forming under the CMV promoter (Kendall et al., 2018). Similarly, the FP-RMS Cre/LoxP mouse model developed tumors in an embryonic/fetal myoblast lineage, but tumor penetrance changed based on when expression was initiated during myogenic development (Abraham et al., 2014). Fusion oncogene expression in mouse endothelial lineages is also permissive to FP-RMS and had similar transcriptional and epigenetic profiles to those of the myoblast lineage (Searcy et al., 2023). Many lineages are intolerant of PAX3::FOXO1 expression with tumors never forming, highlighting the challenges in creating new animal models. Cre/LoxP-driven mouse synovial sarcoma (SS) most readily forms in a *Myf5*-expressing skeletal muscle-specific lineage, whereas fusion oncogene expression in early myogenic precursors (*Pax3*/*Pax7*) was fatal and myofiber expression (*Myf6*) caused myopathy (Haldar et al., 2007; Haldar et al., 2009; Jones et al., 2016). An osteochondroprogenitor lineage also allowed for SS tumorigenesis (Barrott et al., 2018). In clear cell sarcoma (CCS), the fusion oncogene formed tumors with broad expression but also caused developmental delays (Straessler et al., 2013). Lineage-specific expression by doxycycline suggested that tumors arose primarily from neural crest or mesenchymal lineages (Yamada et al., 2013). Overall, tumor formation in distinct lineages suggests that the cellular context and developmental timing alters access to programs needed for transformation.

Specific mutation requirements seem unlikely to be responsible for lineage susceptibility as fusion-driven pediatric cancers have an overall low mutational burden (Gröbner et al., 2018). As FP-RMS mouse tumors have similar chromatin environments independent of their initial lineage, understanding epigenetic regulation in FP-RMS is a valuable therapeutic opportunity. The BAF chromatin remodeling complex has exchangeable subunits that can alter its function between cell types (Centore et al., 2020). During myogenesis, the main catalytic subunit switches from BRG1 to BRM as cells differentiate (Albini et al., 2015). As a leading cell-of-origin candidate for SS is a myogenic precursor, understanding the necessity of each catalytic subunit would be therapeutically advantageous. For example, *in vitro*, FP-RMS utilizes the BRG1 subunit to keep cells undifferentiated (Laubscher et al., 2021). The activity of developmental signaling pathways also varies between lineages. Examples include Hippo signaling in FP-RMS, Wnt/β-catenin signaling in SS, and MITF expression in CCS. A synthetic lethality approach on primary FP-RMS mouse tumor cells with inhibition of NFκB and the apoptosis regulator BCL2 synergistically repressed cell growth (Cleary et al., 2017). Overall, comparative analysis between susceptible and non-susceptible cellular lineages would identify vital cofactors for tumor formation and targets to promote synthetic lethality.

**Table 1.**
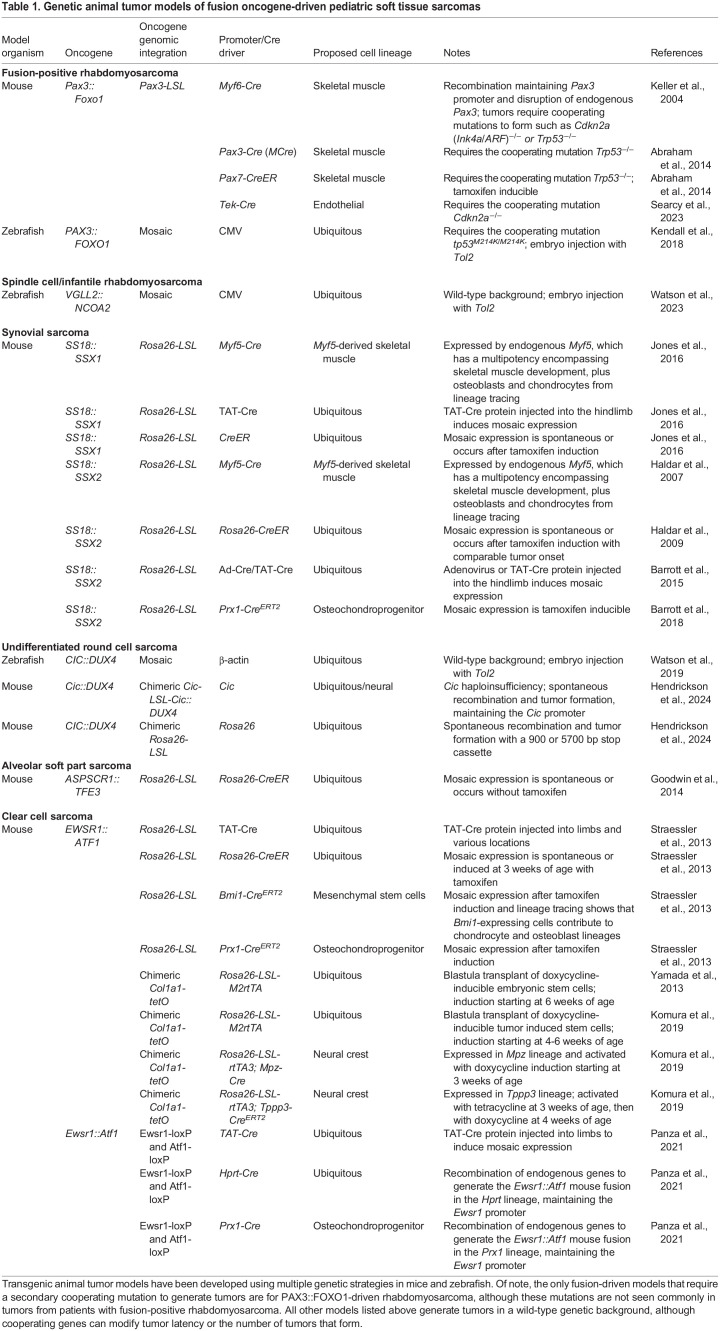
Genetic animal tumor models of fusion oncogene-driven pediatric soft tissue sarcomas

## Fusion-positive RMS driven by PAX3/7::FOXO1 fusions

### Clinical presentation

RMS is the most common pediatric soft tissue sarcoma and has molecular features of abnormal skeletal muscle differentiation (Davicioni et al., 2009; Skapek et al., 2019). The World Health Organization has classified RMS into four histological subtypes: alveolar, embryonal, pleomorphic and spindle cell/sclerosing, all of which have heterogeneous expression of typical skeletal muscle markers (Altmannsberger et al., 1985; Cessna et al., 2001; Choi and Ro, 2021). The two most common RMS subtypes are genetically discriminated by the presence or absence of a gene fusion and include fusion-negative (FN) RMS (embryonal) or fusion-positive (FP) RMS (alveolar). FN-RMS is more common and is genetically driven by RAS, kinase or *TP53* (encoding p53) mutations (Kikuchi et al., 2011; Kim et al., 2021). FP-RMS accounts for 25-30% of all RMS cases and is driven by a chromosomal translocation, which fuses the 5′ region of the gene encoding paired box protein 7 (*PAX7*) on chromosome 1 or paired box protein 3 (*PAX3*) on chromosome 2 to the 3ʹ region of the gene encoding forkhead box protein O1 (*FOXO1*) on chromosome 13 (Barr et al., 1993; Galili et al., 1993; Shapiro et al., 1993; Barr et al., 1996; Rudzinski et al., 2013). PAX3 and PAX7 are paralogs with overlapping, non-redundant functions during neural crest development and myogenesis (Buckingham and Relaix, 2015). FOXO1 is a member of the forkhead transcription factor family, a group that regulates development and myogenesis, maintains homeostasis, and has multiple known roles in cancer (Golson and Kaestner, 2016; Bois and Grosveld, 2003).

The PAX3::FOXO1 fusion occurs in 60-70% of FP-RMS cases, whereas 10-20% have the PAX7::FOXO1 fusion (Raze et al., 2023). Here, FP-RMS refers to the cases driven by PAX3/7::FOXO1 fusions, although other RMS subtypes, such as spindle cell RMS, are driven by fusions as well (Dehner et al., 2023). These fusion oncoproteins retain the full DNA-binding domains of the PAX3/7 transcription factors and transactivation domain of the FOXO1 transcription factor (Fig. 1) (Barr et al., 1993; Galili et al., 1993; Shapiro et al., 1993; Barr et al., 1996). FP-RMS tumors typically present in the extremities with an onset during later adolescence and are more aggressive than the FN-RMS subtype. Patients with FP-RMS have a 5-year survival of around 50%, a greater risk of metastatic disease and a metastatic disease survival under 20% (Heske et al., 2021; Raze et al., 2023; Sorensen et al., 2002). However, for reasons that are not clear, patients with the *PAX7::FOXO1* translocation have a less aggressive disease than patients with the *PAX3::FOXO1* translocation (Missiaglia et al., 2012; Heske et al., 2021; Raze et al., 2023). The current treatment regimen includes surgery, radiation and chemotherapy (vincristine, actinomycin D and cyclophosphamide), to which FP-RMS is initially responsive, but then frequently recurs (Dantonello et al., 2013; Malempati and Hawkins, 2012). Therefore, there is a critical need for targeted therapies informed by the mechanisms of the PAX3/7::FOXO1 fusion oncoproteins.

### Animal tumor models

The original FP-RMS mouse model was developed by incorporating a Cre/LoxP conditional knock-in allele harboring the *Pax3::Foxo1* fusion into the endogenous *Pax3* locus in the *Myf6* lineage, which is highly expressed in postnatal skeletal muscle, but also demarcates progenitor myogenic populations during development (Keller et al., 2004). The *Pax3* allele was converted into *Pax3::Foxo1* when crossed to mice carrying *Myf6-Cre*. The initial cross resulted in tumor formation in one out of 228 mice. However, when mice were crossed in either *Trp53* (encoding p53 in mice) or *Cdkn2a* (also known as *Ink4a* or *ARF*) knockout (−/−) backgrounds, tumor penetrance increased. These mutations disrupt the p53 pathway, but the exact mechanisms and role of p53 in FP-RMS remains unclear. The mouse models in both mutational backgrounds transcriptionally and histologically recapitulated the human disease and presented with aggressive disease characteristics such as metastasis (Nishijo et al., 2009). This system was also used to drive *Pax3::Foxo1* expression in embryonic muscle [under the *Pax3-Cre* (*MCre*) promoter], embryonic and fetal muscle (under *Myf6-Cre*), or postnatal satellite cells (under *Pax7-CreER*); overall, the embryonic and fetal muscle lineage had the highest penetrance and quickest tumor onset (Abraham et al., 2014). This finding agrees with single-cell RNA-sequencing (scRNA-seq) experiments in orthotopic mouse models, which showed that FP-RMS cells exist in a fetal-to-embryonic transitional cell state (Wei et al., 2022).

To begin understanding context-specific effects of the PAX3::FOXO1 fusion, primary mouse tumor cells from each lineage were treated with epigenetic inhibitors. After treatment, *Pax3::Foxo1* expression was repressed in every cell line except those derived from the satellite cell lineage (Abraham et al., 2014). Additionally, later work saw that *Myf6-Cre Pax3::Foxo1* mouse tumors heterogeneously expressed the fusion oncogene (Regina et al., 2021). Isolated tumor cells with lower *Pax3::Foxo1* expression had a higher oncogenic capability in allograft models and more migratory potential *in vitro*, a phenomenon that is also seen for the *EWSR1::FLI1* fusion in Ewing sarcoma (Franzetti et al., 2017; Seong et al., 2021). Altogether, this work demonstrates that lineage and intratumor heterogeneity can impact oncogenicity.

Recently, work using the *Pax3::Foxo1* mouse model with a Tek-Cre driver showed that restricting expression to an endothelial lineage formed RMS tumors (Searcy et al., 2023). *Pax3::Foxo1* expression in other endothelial lineages, driven by either *aP2-Cre* or *Fabp4-Cre*, were also tumorigenic, but mainly developed non-RMS tumors (Searcy et al., 2023). Together, these mouse models indicate that *Pax3::Foxo1* is tumorigenic in multiple lineages, but exact outcomes can vary between context and lineages.

Zebrafish are a complementary vertebrate model for studying sarcomas. Some advantages include nimble genetic modeling for different developmental stages/contexts of tumorigenesis while testing and discovering novel therapeutic approaches (Patton et al., 2021; Kent et al., 2024; Kendall and Amatruda, 2016). Mosaic integration of human *PAX3::FOXO1* into the zebrafish genome generated tumors that recapitulated the human disease with an average onset of around 200 days (Kendall et al., 2018). In this system, *PAX3::FOXO1* was under control of the ubiquitous CMV promoter and was introduced into single-cell embryos using DNA construct microinjection with *Tol2* transposase. PAX3::FOXO1 expression was mosaic due to the delay in *Tol2* translation and random genomic incorporation of the transgene. As in the mouse model, zebrafish require disruption of *tp53* for tumorigenesis; however, in this case, it was a missense mutation in the DNA-binding domain that was predicted to cause gain-of-function activity (Berghmans et al., 2005). This model was used to identify the neural transcription factor HES3 as a novel PAX3::FOXO1-cooperating gene by leveraging zebrafish and cell culture approaches.

### Mechanisms and therapeutic opportunities

#### PAX3::FOXO1

PAX3::FOXO1 is a transcriptional activator with pioneering activity (Sunkel et al., 2021). Pioneer factors are a distinct class of transcription factors that bind to inaccessible and generally silent chromatin, which allows them to set or change cell fate decisions (Sunkel and Stanton, 2021). Yet, pioneer factor binding sites vary between cell contexts, and pioneer factor binding precedes gene expression, showing that multiple elements could modulate pioneering activity (Cernilogar et al., 2019; Donaghey et al., 2018; Gualdi et al., 1996). These elements suggest that cofactor availability between susceptible and non-susceptible tumorigenic lineages could modulate PAX3::FOXO1 activity. PAX3::FOXO1 colocalizes with myogenic transcription factors and chromatin remodelers on DNA (Laubscher et al., 2021; Gryder et al., 2017; Bharathy et al., 2022; Marques et al., 2020). PAX3::FOXO1 also directly interacts with the p300 (encoded by *EP300*) histone acetyltransferase and utilizes the CBP (encoded by *CREBBP*)/p300 coactivator complex through the FOXO1 activation domain (Asante et al., 2023). Altering histone acetylation hinders FP-RMS cell and xenograft growth, but xenograft growth inhibition is dependent on a particular dosing schedule (Gryder et al., 2019; Bharathy et al., 2018; Kurmasheva et al., 2019; Pacenta et al., 2021). In a phase I clinical trial for relapsed RMS, a combination of the histone deacetylase (HDAC) inhibitor mocetinostat and microtubule inhibitor vinorelbine had a favorable safety profile, and six out of seven evaluated patients had a partial response or stable disease (Federman et al., 2022). Testing HDAC inhibitor combination therapies and inhibitors of other chromatin regulators in genetic animal models could allow further assessment of efficacies, mechanisms of action and developmental toxicities.

scRNA-seq datasets have highlighted distinct cell states in FP-RMS xenograft models, which include a neuronal cell population (Wei et al., 2022; Danielli et al., 2023a; Patel et al., 2022). Despite being, in part, histologically diagnosed by expression of myogenic markers, transcriptional data from patients with FP-RMS show enrichment for neural-related pathways (Raghavan et al., 2019). Moreover, an *in ovo* model electroporated stage 11 chicken embryos and showed that PAX3::FOXO1 trans-differentiated neural cells into RMS-like cells (Gonzalez Curto et al., 2020). These cells gained myogenic phenotypes and expressed markers of epithelial-mesenchymal transition and cell migration, and displayed tissue-invasion properties. This trans-differentiation supports the hypothesis that the neuronal lineage may be a susceptible population for FP-RMS tumorigenesis.

The neuronal cell subpopulation identified by scRNA-seq in xenografts from patients with FP-RMS is also resistant to a frequently used chemotherapeutic combination of vincristine and irinotecan, suggesting a functional role for neural pathways in disease progression (Danielli et al., 2023b preprint). In zebrafish embryos injected with *PAX3::FOXO1*, one of the most upregulated genes is *her3*, the zebrafish ortholog to *HES3* (Hatakeyama et al., 2004; Kendall et al., 2018). Knockout of *her3* in zebrafish results in transcriptional inhibition of matrix metallopeptidases, suggesting that HES3 could modulate FP-RMS migration or invasion (Kent et al., 2023). Incorporation of this *her3* mutant zebrafish line into the zebrafish FP-RMS model will further elucidate HES3 contributions to tumorigenesis. The Hippo/MST1 tumor suppressor pathway, which regulates neural crest and nervous system development, also has an important cooperating role in FP-RMS. PAX3::FOXO1 inhibits the Hippo/MST1 signaling pathway, and genetic removal of Mst1/Mst2 Hippo pathway members in the *Myf6-Cre Pax3::Foxo1* mouse model increased the frequency of head and neck tumors (Sahu and Mondal, 2021; Crose et al., 2014; Oristian et al., 2018). Altogether, the prevalence of a neural cell population and neural pathway activation justifies further investigation; it would be valuable to mechanistically investigate how PAX3::FOXO1 differentially cooperates with neural factors versus with classical myogenic markers.

#### PAX7::FOXO1

Most FP-RMS animal models have focused on the *PAX3::FOXO1* fusion oncogene, given its significantly worse prognosis for patients. However, developing *PAX7::FOXO1* tumor models would allow for comparative analyses to identify mechanisms responsible for disease aggressiveness. PAX7::FOXO1 has been modeled in *Drosophila melanogaster* (Galindo et al., 2006)*.* Leveraging the UAS/GAL4 system, human *PAX7::FOXO1* is expressed in myosin heavy chain-positive cells (Galindo et al., 2006; Brand and Perrimon, 1993). Here, *PAX7::FOXO1* expression results in disorganized skeletal muscle cells that invade the central nervous system, which typically lacks myogenic tissue. Transcriptional profiling and genetic screening focused on the lethality of *PAX7::FOXO1* found that the myogenic regulators *Mef2* (in *Drosophila*) and *Tanc1*/*rols* (in mouse C2C12 cells and *Drosophila*, respectfully) affected PAX7::FOXO1 function, highlighting the importance of myogenic factors in this disease (Galindo et al., 2014; Avirneni-Vadlamudi et al., 2012).

To directly compare PAX3::FOXO1 and PAX7::FOXO1 fusion oncoprotein activity, one study ectopically expressed *PAX3::FOXO1* or *PAX7::FOXO1* in fibroblasts (Manceau et al., 2022). Each fusion protein had slightly different DNA motif preferences, and PAX7::FOXO1 had a more robust transcriptional activation potential as it highly colocalized with *de novo* deposition sites of the active histone mark H3K27ac (acetylation of histone 3 at lysine 27) (Manceau et al., 2022; Du et al., 2005). Transcriptionally, PAX3::FOXO1 activated genes associated with the cell cycle, cell migration and metabolism, whereas PAX7::FOXO1 altered embryonic lineage differentiation and cytoskeletal remodeling pathways, which caused more severe changes to cell morphology. Therefore, the regulatory activities between these fusions could explain their clinical differences.

## Infantile/spindle cell RMS driven by VGLL2::NCOA2 fusion

### Clinical characteristics

Infantile or spindle cell/sclerosing RMS is another subtype that is genetically driven by gene fusions. Fusions contain rearrangements with genes encoding either vestigial-like family member 2 (*VGLL2*) or nuclear receptor coactivator 2 (*NCOA2*), including *VGLL2::NCOA2* (Alaggio et al., 2016), *VGLL2::CITED2* (Alaggio et al., 2016), *SRF::NCOA2* (Mosquera et al., 2013) and *TEAD1::NCOA2* (Mosquera et al., 2013) (Fig. 1). Although all of these fusion genes are thought to be transforming, *VGLL2::NCOA2* is the only fusion with a genetic animal model confirming its oncogenic capacity (Watson et al., 2023). VGLL2 partially functions by interacting with the TEAD transcription factor family, which is a downstream effector of the Hippo pathway, whereas NCOA2 is part of the p160 coactivator family (Maeda et al., 2002; Chen et al., 2004; Voegel et al., 1996; Hong et al., 1997). *VGLL2::NCOA2* arises from two genetic events: an inversion of exons 2 or 3 of *VGLL2*, and a translocation between chromosomes 6 and 8 to juxtapose *VGLL2* exons 2 or 3 to NCOA2 exons 13 or 14 (Alaggio et al., 2016; Watson et al., 2018) (Fig. 1).

Infantile RMS usually presents in the first year of life and is mostly found on the chest wall, back, neck and arm (Mosquera et al., 2013; Alaggio et al., 2016; Cyrta et al., 2021; Butel et al., 2020). Initial cases suggested that *VGLL2-*rearranged RMS had a more favorable prognosis; however, recently, four patients with *VGLL2-*rearranged RMS experienced local progression and metastasis, with half of these children succumbing to their disease (Cyrta et al., 2021). These recent reports have underscored that the *VGLL2* fusion oncogene can be aggressive and is not always an indolent disease, highlighting the need for targeted therapies, as patients normally undergo surgery and general chemotherapy (Cyrta et al., 2021; Butel et al., 2020) .

### Animal tumor models

To investigate the tumorigenic capacity of *VGLL2::NCOA2*, a transgenic zebrafish model was developed. In this model, the human *VGLL2::NCOA2* coding sequence was incorporated into the zebrafish genome under the ubiquitous CMV promoter using a similar approach to the *PAX3::FOXO1*-driven zebrafish tumor model (Watson et al., 2023; Kendall et al., 2018). Tumors formed within 6 months and did not require a sensitizing mutation. To perform a cross-species comparative oncology approach, *VGLL2::NCOA2* was transfected into mouse myoblast cells and allografted into immunocompromised mice. RMS mouse allograft tumors rapidly developed, and both mouse allograft and transgenic zebrafish tumors transcriptionally and histologically recapitulated the human disease. RNA sequencing in tumors from both species showed that VGLL2::NCOA2 reactivated developmental programs and displayed molecular features of abnormal skeletal muscle development (Watson et al., 2023). The small GTPase ARF6 was also functionally evaluated *in vitro* due to its overexpression across models and patient tumors compared to its expression in mature skeletal muscle. *Arf6* knockout in mouse C2C12 cells significantly reduced VGLL2::NCOA2-directed colony formation, thus signifying it as a potential therapeutic vulnerability (Watson et al., 2023).

### Mechanisms and therapeutic opportunities

Understanding the normal functions of both VGLL2 and NCOA2 fusion partners may provide insights into the tumorigenic functions of the VGLL2::NCOA2 fusion oncoprotein. As a skeletal muscle-specific coactivator, VGLL2 is expressed in differentiating somites and adult skeletal muscle tissue (Mielcarek et al., 2002; Maeda et al., 2002). VGLL2 contains a tondu (TDU) domain that interacts with TEAD family transcription factors for skeletal muscle differentiation (Maeda et al., 2002; Chen et al., 2004; Gunther et al., 2004; Honda et al., 2017). As the *TEAD1::NCOA2* fusion gene is also a genetic driver in infantile RMS, this suggests convergent roles for TEAD-dependent pathways in this disease (Tan et al., 2020; Alaggio et al., 2016; Mosquera et al., 2013).

*NCOA2* is a common 3′ fusion partner in many cancers (Wang et al., 2012; Strehl et al., 2008; Zhuravleva et al., 2008). An *in vitro* investigation of *PAX3::NCOA2*, another RMS fusion oncogene, determined that deletion of the CID/AD1 domain in *NCOA2* reduces colony formation (Sumegi et al., 2010). The transformation capability of the CID/AID1 domain is, in part, due to its interaction with the histone acetyltransferases CBP and p300 (Deguchi et al., 2003). As this domain is retained in the VGLL2::NCOA2 protein, it would be interesting to understand how VGLL2::NCOA2 alters CBP or p300 activity to promote tumorigenesis.

## SS driven by SS18::SSX fusion

### Clinical presentation

SS is the second most common pediatric soft tissue sarcoma, with 30% of cases occurring in patients under 20 years of age (Palmerini et al., 2009; Aytekin et al., 2020; Gazendam et al., 2021). SS is a slow-growing mass that arises in the soft tissue around the knees and ankles (Gazendam et al., 2021). The overall survival for localized disease is 70%, whereas metastatic disease has a 5-year overall survival under 20% (Aytekin et al., 2020; Palmerini et al., 2009). Metastatic disease ultimately develops in 50-70% of cases (Krieg et al., 2011; Singer et al., 1996; Vlenterie et al., 2016). The standard of care is surgical resection with radiotherapy or chemotherapy, but effectiveness of these therapies remains unclear (Von Mehren et al., 2016; Gazendam et al., 2021).

The genetic hallmark of SS is a chromosomal translocation between *SS18* on chromosome 18 and either *SSX1*, *SSX2* or *SSX4* on the X chromosome (Fig. 1) (Limon et al., 1986; Clark et al., 1994; Crew et al., 1995; de Leeuw et al., 1995). SS18 is a subunit of the ATP-dependent BAF chromatin remodeling complex that moves or evicts histones to modulate gene expression, and SSX proteins are transcriptional repressors (Alfert et al., 2019; Ladanyi, 2001). *SS18::SSX1* and *SS18::SSX2* are the most common fusions and it is unclear whether patient outcomes differ based on which SSX protein is in the fusion (Kubo et al., 2015). In SS18::SSX, the SS18 C-terminal domain is replaced by the final 78 amino acids of SSX1, SSX2 or SSX4, but the repression domain of SSX proteins is not retained (Clark et al., 1994; Ladanyi, 2001). The SS18::SSX fusion oncoprotein causes transcriptional dysregulation by altering the activity of the BAF complex and other chromatin regulatory complexes (Kadoch and Crabtree, 2013).

### Animal tumor models

An SS mouse model was developed by inducing *SS18::SSX2* expression under the *ROSA* promoter in the presence of Cre recombinase (Haldar et al., 2007). When these mice were crossed with *Myf5-Cre* mice, *SS18::SSX2* was expressed in a skeletal muscle-specific lineage. There was early lethality in 8% of the *Myf5-Cre* progeny, but the remaining mice generated SS between 3 and 5 months of age. A similar approach was used to develop the *SS18::SSX1* mouse model, which is slightly less oncogenic than the *SS18::SSX2* model (Jones et al., 2016). To avoid embryonic toxicity, the authors modified their approach and used tamoxifen-inducible CreER to express the fusion oncogene at 3 months of age (Haldar et al., 2009). CreER-driven tumor onset was delayed to 5-14 months, but mice tended to have more individual tumors. Additionally, *SS18::SSX2* expression in earlier or later myogenic lineages resulted in lethality or myopathy, highlighting the need for a particular lineage for tumorigenesis and suggesting a multipotent mesenchymal stem cell origin. However, the identity of the exact lineage(s) of origin remains debated in the field.

### Mechanisms and therapeutic opportunities

Animal modeling has connected various oncogenic factors and pathways to SS tumorigenesis (reviewed by Landuzzi et al., 2023). From these models, it is clear that a specific cell state is required to tolerate *SS18::SSX* expression; yet, how does one particular cell type survive a fusion oncoprotein that contains a ubiquitous and essential BAF complex protein? A potential explanation is that the BAF complex has interchangeable components that can form three main subcomplexes – canonical BAF (cBAF), polybromo BAF (pBAF) or non-canonical BAF (ncBAF) – that can alter the activity of the complex (reviewed by Centore et al., 2020). The BAF complex with SS18::SSX can differentially activate targets depending on the *in vitro* cell type (Tamaki et al., 2015). *In vivo Smarcb1* (also known as *Baf47*) knockout *Myf5-*Cre *SS18::SSX2* mice had a higher disease penetrance, shorter latency and reduced BAF complex genomic binding (Li et al., 2021). The presence of SS18::SSX2 destabilized cBAF and altered the overall composition of the BAF subcomplexes. This potential dependency on the other BAF subcomplexes could be targeted by BRD9 inhibition, which is distinct to ncBAF (Brien et al., 2018; Michel et al., 2018). As such, BRD9 protein degraders, which mark targets for degradation by the endogenous ubiquitin ligase system, have begun phase I clinical trials for various cancers.

SS18::SSX also alters the interaction between the BAF and the Polycomb repressive complexes, as BAF opposes Polycomb transcriptional repression (Kadoch et al., 2017). SS18::SSX can co-occupy DNA with the Polycomb repressive complex variant PRC1.1, which deposits the repressive histone mark H2AK119ub (monoubiquitination of histone 2A at lysine 119) (McBride et al., 2020; Banito et al., 2018). This fusion protein interaction is driven by the C-terminus of SSX1 and localizes SS18::SSX to sites with H2AK119ub1, further stabilizing PRC1.1 on DNA to promote the deposition of this histone mark *in vivo* (Benabdallah et al., 2023). SS18::SSX organoids also recruit PRC1.1 and alter H2AK119ub deposition (Boulay et al., 2021). Together, these works suggest that targeting PRC1.1 could be an alternative therapeutic approach.

FGFR signaling is another important *in vivo* signaling pathway for SS (DeSalvo et al., 2021). Using a triple mutant *Fgfr1*/*Fgfr2*/*Fgfr3* mouse crossed with the *Myf5-Cre SS18::SSX2* mouse model, one study tested FGFR knockout mice as single, double and triple knockouts; each mutant combination could repress tumor penetrance (DeSalvo et al., 2021). Pharmacological inhibition of FGFR signaling also suppressed tumorigenesis, prevented MAPK signaling and downregulated the expression of the ETV4 and ETV5 transcription factors, which were overexpressed in human and mouse tumor samples. *In vitro*, ETV4 or ETV5 knockdown upregulated the DUX4 transcription factor and promoted cell death. Altogether, this work highlights how FGFR inhibition could be a promising strategy for clinical evaluation in a combination therapy.

Another pathway implicated in SS is the Wnt signaling pathway, which regulates cell proliferation and differentiation, and is frequently dysregulated in cancer (Liu et al., 2022). Tumors from patients with SS have high levels of nuclear staining for β-catenin (encoded by *CTNNB1*; a marker of active Wnt signaling) (Horvai et al., 2006). In the SS18::SSX2 mouse model, silencing β-catenin suppressed tumor formation (Barham et al., 2013). This result was recapitulated with Wnt pathway pharmacological inhibition without obvious toxicities in mice. A gain-of-function approach with an adenovirus-induced Cre SS mouse model, which stabilized β-catenin in the nucleus, accelerated tumor onset, and tumors had more invasive characteristics (Barrott et al., 2015). In a *Prx1-CreERT2* osteochondroprogenitor lineage-restricted model of SS18::SSX2, tumors formed when β-catenin was stabilized, suggesting that Wnt signaling can modulate cellular susceptibility to the fusion oncogene (Barrott et al., 2018).

Wnt/β-catenin signaling was also activated in an *ex vivo SS18::SSX1* mouse model that showed that SS18::SSX1 non-autonomously cooperated with *miR-214* and upregulated *Il8* (also known as *Cxcl15*) (Tanaka et al., 2020). This finding suggests that SS18::SSX modulates tumor and immune system interactions through Wnt/β-catenin activation and agrees with findings that Wnt/β-catenin signaling broadly correlates with cancer immune system evasion (Luke et al., 2019). Immune-evasive SS cells identified from scRNA-seq tend to be more aggressive (Jerby-Arnon et al., 2021). Therefore, developing effective immunotherapies may require inhibiting Wnt/β-catenin activity. Overall, Wnt/β-catenin signaling impacts multiple SS tumorigenic characteristics, making it a therapeutic target of significant interest, especially for patients with aggressive disease.

## URCS driven by CIC::DUX4 fusion

### Clinical presentation

Initially classified as Ewing sarcoma due to an overlap in cellular morphology and immunohistochemistry, the World Health Organization now classifies capicua transcriptional repressor (*CIC*)-rearranged sarcoma as URCS of soft bone and tissue, given its distinct molecular and transcriptional characteristics (Choi and Ro, 2021; Specht et al., 2014). Most *CIC*-rearranged cases harbor a gene fusion from *CIC* on chromosome 19 to the double homeobox 4 gene (*DUX4*), which resides on either chromosome 4 or chromosome 10 (Fig. 1) (Kawamura-Saito et al., 2006; Yoshimoto et al., 2009; Italiano et al., 2012). CIC was first identified in *Drosophila melanogaster* and is a transcription factor that regulates a range of developmental processes including lung and brain development (Jiménez et al., 2000; Lee et al., 2011; Lu et al., 2017). DUX4 is a transcription factor that is associated with facioscapulohumeral muscular dystrophy and Bosma arrhinia microphthalmia (Hendrickson et al., 2017; Dixit et al., 2007; Bosnakovski et al., 2008; Inoue et al., 2023). The *CIC::DUX4* fusion oncogene encodes the N-terminal and DNA-binding domain of CIC, with the addition of the C-terminus of DUX4, which converts CIC from a transcriptional repressor to an activator (Kawamura-Saito et al., 2006). CIC::DUX4-positive tumors have a distinct gene signature in which the *WT1* transcription factor and members of the ETS transcription factor family (*ETV1*, *ETV4* and *ETV5*) are overexpressed (Specht et al., 2014).

In a study of 115 patients with *CIC*-rearranged sarcomas, the age at diagnosis ranged from 6 to 81 years, with 22% of patients being under the age of 18 (Antonescu et al., 2017). The presentation of the tumor predominantly occurred in the trunk or extremities and displayed a diverse morphology that included spindle, epithelioid and round cells. Although patients with localized and fully resected URCS have a 5-year survival of around 50%, patients with metastatic disease have an overall survival of under 2 years (Antonescu et al., 2017; Brahmi et al., 2023). The standard of care includes surgical resection and chemotherapeutic regimens similar to those for Ewing sarcoma, with additional radiation for advanced disease (Connolly et al., 2022).

### Animal tumor models

A CIC::DUX4-driven animal tumor model was developed in zebrafish using a similar approach to that for the RMS models (Watson et al., 2019 preprint; Kent et al., 2024; Kendall et al., 2018; Watson et al., 2023). Here, the human *CIC::DUX4* coding sequence was driven by the zebrafish β-actin promoter (Watson et al., 2019 preprint). This model had an average tumor onset of around 75 days and a 30% penetrance. Tumors recapitulated human URCS histologically and transcriptionally, which included upregulation of hallmark disease genes, such as *etv1*, *etv4*, *etv5a* and *etv5b*. CRISPR knockout of *etv4* significantly suppressed tumor formation, suggesting that ETV4 has a cooperative role in CIC::DUX4 tumorigenesis.

Multiple mouse models of *CIC::DUX4-*driven sarcoma have been recently developed, each with spontaneous CIC::DUX4 expression (Hendrickson et al., 2024). One model integrated the coding sequence for the C-terminal activation domain of human DUX4 into the endogenous mouse *Cic* gene to match the *Cic* haploinsufficiency seen in patients. Embryonic stem cell clones positive for this alteration were injected into host mice to generate chimeric pups. Although this was under a Cre/LoxP system to control *Cic::DUX4* expression, spontaneous recombination resulted in tumors forming at 3 weeks of age with near complete penetrance by 5 weeks. In an alternative Cre/LoxP approach, *CIC::DUX4* was placed under the ubiquitous *Rosa26* promoter. Tumors spontaneously formed when the pups were 3 weeks old, suggesting that *Cic* haploinsufficiency did not contribute to tumorigenesis. By performing chromatin immunoprecipitation followed by sequencing (ChIP-seq) in an HA-tagged version of the *Rosa26-CIC::DUX4* mouse model cell lines, CIC::DUX4 was found to directly activate ETV transcription factors, as well as the Ras and Wnt signaling pathways. CIC::DUX4-bound sites were enriched with CIC and ETV transcription factor-binding motifs, suggesting that not only did CIC::DUX4 activate ETV transcription factors, but it could also colocalize with them on DNA to drive transcription (Hendrickson et al., 2024). These animal models provide a powerful opportunity to study CIC::DUX4 core circuitry during tumor initiation and in established tumors.

### Mechanisms and therapeutic opportunities

Inhibiting activation of CIC::DUX4 gene targets, such as ETV4, may be a potential therapeutic approach. In an orthotopic mouse model, *Etv4* knockdown reduced tumor growth and metastasis, suggesting a role in more aggressive disease (Okimoto et al., 2019). *In vitro*, ChIP-seq of CIC::DUX4 indicated that the fusion oncoprotein highly colocalizes with the active histone mark H3K27ac (Thomas et al., 2023 preprint). Gene activation by CIC::DUX4 requires CBP and/or p300, as drug inhibition of CBP and p300 reverses the regulatory circuitry of the disease, alters the expression of oncogenic pathways and reduces xenograft tumor burden (Bosnakovski et al., 2021).

In URCS, CIC::DUX4 activates various negative MAPK regulators, making the MAPK and PI3K/AKT pathway proteins of clinical interest as drug targets (Lin et al., 2020; Thomas et al., 2023 preprint; Carrabotta et al., 2022). Genetic and pharmacological inhibition of the ERK negative regulator DUSP6 promotes cell death *in vitro*, and *in vivo* pharmacological inhibition reduces tumor burden in a mouse xenograft model (Lin et al., 2020). This result could be due to activated ERK binding to CIC::DUX4 to facilitate its degradation. This suggests that CIC::DUX4 and MAPK pathway interactions function as a feedback loop that modulates CIC::DUX4 expression and could affect intratumor heterogeneity and oncogenicity. Xenografts and cell lines derived from patients with CIC::DUX4-driven sarcomas have hyperactivation of the PI3K/AKT pathway through autocrine signaling by high-mobility group A (HMGA) and insulin-like growth factor (IGF) proteins (Carrabotta et al., 2022). A dual PI3K/AKT and mTOR inhibitor in combination with trabectedin was required to repress tumor growth and metastasis, indicating that combination therapies might be required to see a therapeutic benefit.

CIC::DUX4 also upregulates cell cycle regulators in a chicken chorioallantoic membrane and *ex vivo* mouse model (Komatsu et al., 2021; Yoshimoto et al., 2017). Cyclins and cyclin-dependent kinases (CDKs) drive and regulate cell division, which can become hyperactive in cancers (Fassl et al., 2022). In the *ex vivo* mouse model, *Ccnd2*, or cyclin D2, was found as a disease marker. *In vitro*, *Ccnd2* knockdown suppressed cell growth and, *in vivo*, pharmacological inhibition with palbociclib, a CDK4/CDK6 inhibitor, or trabectedin repressed tumor growth of mouse URCS (Yoshimoto et al., 2017). In the orthotopic mouse metastasis model, *Ccne1*, or cyclin E1, was upregulated, and pharmacological inhibition of CDK2 repressed tumor growth and metastasis (Okimoto et al., 2019). Broadly, CDK inhibitors have shown promise in sarcomas, but further testing as monotherapies and combination therapies in animal models would better establish their efficacy in URCS (Hsu et al., 2022).

## ASPS driven by ASPSCR1::TFE3 fusion

### Clinical presentation

ASPS histologically resembles lung alveoli and occurs in soft tissues of the extremities and trunk regions (Christopherson et al., 1952; Wang et al., 2016; Fujiwara et al., 2023; Cloutier and Charville, 2019). Representing under 1% of all soft tissue sarcomas, ASPS is often diagnosed between 15 and 35 years of age (Zhang et al., 2022; Wang et al., 2016; Fujiwara et al., 2023). ASPS is a slow-growing tumor at diagnosis and generally lacks symptoms, resulting in most patients presenting with metastatic disease (O'Sullivan Coyne et al., 2021; Fujiwara et al., 2022). Overall survival for patients with metastatic disease ranges from 20 to 62% across various studies, whereas patients with localized disease have an overall survival ranging from 60 to 88% (Fujiwara et al., 2023). The primary treatment for localized disease is surgery, in which complete resection can be curative, but metastasis remains common (Chang et al., 2021). ASPS is largely resistant to standard radiotherapy and chemotherapy approaches (Fujiwara et al., 2023).

The hallmark genetic driver of ASPS is a chromosomal translocation between chromosome 17 and the X chromosome, which generates the *ASPSCR1::TFE3* fusion gene (Fig. 1) (Heimann et al., 1998; Joyama et al., 1999; Mitton and Federman, 2012). TFE3 is part of the MiT/TFE3 transcription factor family, which regulates processes involved in cellular stresses, and ASPSCR1 is involved in insulin regulation in muscle and fat cells (La Spina et al., 2020; Bogan and Kandror, 2010). The fusion oncoprotein functions as an aberrant transcription factor that regulates autophagy and angiogenesis-related genes with stronger transcriptional activation activity than that of TFE3 (Kobos et al., 2013; Stockwin et al., 2009; Lazar et al., 2007). The development of new treatment strategies has primarily revolved around the angiogenic properties of this disease and immunotherapies (Brahmi et al., 2020).

### Animal tumor models

Using the Cre/LoxP system, a mouse model of ASPS was developed to drive conditional *ASPSCR1::TFE3* expression (Goodwin et al., 2014). This mouse model expressed *ASPSCR1::TFE3* at the ubiquitous *Rosa26* gene locus and used CreER, without tamoxifen, to spontaneously and at low levels induce *ASPSCR1::TFE3* expression across tissues. Although these mouse ASPS tumors were histologically and transcriptionally consistent with the human disease, they had a unique presentation in the skull instead of the skeletal muscle (the presentation that is seen in patients). Mouse tumor presentation remained consistent even when *ASPSCR1::TFE3* was expressed under a tissue-specific promoter. To explain the cranial presentation of the tumors, the authors noted that both mouse and human tumors had high expression of lactate transporters and could rapidly metabolize lactate. In patient-derived cell lines, *in vitro* lactate treatment enhanced proliferation and vascularization. Both brain and skeletal muscle have high lactate levels, suggesting that lactate could be a driver of tumorigenesis in patients.

### Mechanisms and therapeutic opportunities

Immunotherapies have shown promise in a select number of patients treated for ASPS (Fujiwara et al., 2023). In a study for the PDL1 (encoded by *CD274*) inhibitor atezolizumab, 37% of the 52 patients had a response to treatment, with an average progression-free survival of almost 21 months (Chen et al., 2023). However, questions remain as to why some patients were unresponsive. Potentially, this could be due to the high lactate tumor microenvironment as lactate can induce immunosuppression and promote tumor immune evasion (Li et al., 2022b). Therefore, studying immunotherapies in the ASPS genetic mouse model could identify immunosuppression and resistance mechanisms or test combination therapies to enhance immunotherapy efficacy.

The highly vascularized nature of ASPS tumors makes targeting angiogenesis pathways of interest. An *ex vivo* ASPS mouse model generated vascularized tumors with complete penetrance and metastatic disease (Tanaka et al., 2017). ASPSCR1::TFE3 ChIP-seq on mouse tumor cells and patient-derived cells determined that the fusion oncoprotein highly colocalized with the active histone mark H3K27ac and bound near genes related to blood vessel development (Tanaka et al., 2023). A CRISPR/dCas9 screen of *ex vivo* tumor cells before transplantation focused on reducing activity of select oncogenic enhancers and identified several genes involved in angiogenic trafficking pathways and vascular regulation, such as *Rab27a*. RAB27A is a GTPase that stabilizes VEGFR1 (encoded by *FLT1*), a member of the endothelial growth factor receptor family that modulates vascular morphology (Boucher et al., 2017). This pathway could be a target for further evaluation in animal models because pazopanib, a tyrosine kinase inhibitor targeting VEGF receptors, was seen to improve progression-free survival in early clinical trials (van der Graaf et al., 2012; Kim et al., 2019).

Epigenetic activation of targets by ASPSCR1::TFE3 appears vital for tumorigenesis. Broadly suppressing effects of histone acetylation by targeting the epigenetic reader BRD4 with the small-molecule inhibitor JQ1 repressed angiogenesis-related gene expression and inhibited *ex vivo* tumor formation (Tanaka et al., 2023). Additionally, a recent publication found that ASPSCR1::TFE3 and VCP (also known as p97), an ATPase that can modulate protein membrane and complex interactions, directly interact in the genetic ASPS mouse model and patient cell lines (Pozner et al., 2024; Meyer and Weihl, 2014). VCP organized enhancer-promoter interactions and supported oncogenic gene expression, and pharmacological inhibition of VCP repressed xenograft tumor growth, presenting a potential new therapeutic avenue.

## CCS driven by EWSR1::ATF1 fusion

### Clinical presentation

CCS has melanocytic characteristics and may arise from neural crest cells (Ibrahim et al., 2018; Kindblom et al., 1983). CCS occurs in teenagers and young adults, with an average age of diagnosis around 25 years and a presentation often in the lower extremities near tendons and aponeuroses (Eckardt et al., 1983; Deenik et al., 1999; Kuiper et al., 2003; Gonzaga et al., 2018; Enzinger, 1965). Patients frequently present with metastatic disease and there is a high rate of recurrence. The overall survival is 50% and 38% for 5 and 10-year periods, respectively (Gonzaga et al., 2018). Currently, surgical resection is the primary treatment because CCS is minimally responsive to chemotherapy and radiation (Ibrahim et al., 2018).

CCS was originally classified as malignant melanoma, but the discovery of a fusion oncogene differentiated CCS into its own distinct subtype (Enzinger, 1965; Ibrahim et al., 2018). The primary chromosomal translocation occurs between chromosome 12 and chromosome 13 in 80-90% of cases and generates the *EWSR1::ATF1* fusion oncogene (Fig. 1) (Panagopoulos et al., 2002; Zucman et al., 1993; Rivera et al., 2022; Kobayashi et al., 2023). Ewing sarcoma RNA-binding protein 1 (EWSR1) is a ubiquitously expressed protein with RNA-binding activity and broadly regulates cellular processes (Lee et al., 2019). Activating transcription factor 1 (ATF1) is part of the cAMP response element-binding protein family and responds to extracellular signals to maintain homeostasis (Chen et al., 2022). The fusion oncoprotein functions as a transcription factor that binds to DNA sequences similar to those bound by ATF1, but with enhanced gene-activation capabilities (Jishage et al., 2003; Fujimura et al., 1996; Brown et al., 1995).

### Animal tumor models

The earliest animal models of CCS suggested that the disease requires a distinct cellular state to promote tumorigenesis. One genetic mouse model used multiple variations of the Cre/LoxP system to drive *EWSR1::ATF1* expression (Straessler et al., 2013). Ubiquitous *EWSR1::ATF1* induction by either TAT-Cre or tamoxifen (for CreER) injection resulted in tumor formation and additional developmental deformities. Lineage-specific Cre promoters caused various outcomes. EWSR1::ATF1 expression in 3-week-old mice driven in early mesenchymal lineages by either *Prx1-CreER* or *Bmi1-CreER* generated tumors at approximately 8 weeks with complete penetrance.

CCS lineages of origin have also been investigated with a doxycycline-inducible *EWSR1::ATF1* mouse model (Yamada et al., 2013). Around 4 weeks post doxycycline induction, the mice developed tumors. Even though fusion oncogene expression was ubiquitous, there was a predilection for tumor presentation in the trunk, with 92% of tumors presenting in that region. Lineage tracing with a *Wnt1* reporter also supported a neural crest progenitor origin as all tumors expressed the reporter construct. Furthermore, when CCS cells were reprogrammed into pluripotent stem cells, *EWSR1::ATF1* expression was induced and ChIP-seq was performed, EWSR1::ATF1 was found to be genomically bound near neural crest genes (Komura et al., 2019). Upon cell injection and doxycycline induction in mice, sarcomas formed in soft tissues near the peripheral nerve wall. Finally, when *Tppp3-Cre*, another neural crest lineage, drove fusion oncogene expression, CCS tumors also developed. Together, these animal models suggest a neural crest/mesenchymal lineage for CCS tumorigenesis.

A mouse model was also developed that induced the translocation from the endogenous mouse genes (Panza et al., 2021). Here, *loxP* elements were inserted into intron 7 of *Ewsr1* and intron 4 of *Atf1*. The *Ewsr1::Atf1* translocation was induced by crossing with *Hprt-Cre* or *Prx1-Cre* mice or with TAT-Cre injection into the limb. Tumors developed at a lower frequency but still mimicked CCS transcriptionally and histologically. However, an increased mutational load was seen compared to that in previous CCS models, with genomic alterations occurring near *Mitf* and *Myc*, suggesting that they are cooperating genes in this disease that warranted functional evaluation. In a *Mitf* loss-of-function mutational background, *Ewsr1::Atf1* mouse tumor formation was slightly delayed. Activating *Myc* resulted in more rapid *Ewsr1::Atf1* tumor development, although these tumors did not histologically recapitulate CCS. The transformative capacity of EWSR1::ATF1 across mouse models highlights the need to develop therapies focused on the mechanisms of this fusion oncoprotein and provides tools to understand cooperating genes.

### Mechanisms and therapeutic opportunities

Given the lethality of EWSR1::ATF1 in multiple lineages, it may be worthwhile to investigate methods to promote oncogene-induced senescence and drive the tumor into an unstable and apoptotic state. After *Ewsr1::Atf1* induction, various mouse organs had higher levels of p53, which is a hallmark of oncogene-induced senescence (Komura et al., 2019; Collado and Serrano, 2006). Induction of the *Ewsr1::Atf1* translocation in mice caused deletions around *Cdkn2a* and an amplification around the *Mdm2* locus, which is known to modulate TP53 activity (Jacob et al., 2014; Panza et al., 2021)*.* In induced pluripotent stem cells (iPSCs), EWSR::ATF1 is bound to *Cdkn2a*, suggesting that EWSR::ATF1 directly regulates cellular senescence pathways during early stages of tumorigenesis (Komura et al., 2019).

One potential mechanism for the differential responses to EWSR1::ATF1 between lineages may be its role as a transcription factor. Transcription factor binding can vary between cell types, and EWSR1::ATF1 binding varied between the inducible iPSC cells and patient-derived CCS cells, which was partially driven by a pre-marking of H3K27ac (Komura et al., 2019; Srivastava and Mahony, 2020). Therefore, differences in chromatin accessibility or available cofactors would alter the genes that are activated. EWSR1::ATF1 targets could be differentially regulated based on context. *MITF* is a direct EWSR1::ATF1 target, is required for *in vitro* CCS cell survival and is a CCS histological marker (Li et al., 2003; Davis et al., 2006). However, *Bmi1-CreER* and *Prx1-CreER*-driven CCS mouse tumors had variable MITF expression, suggesting that MITF activity likely varies by context (Straessler et al., 2013). In xenografts, both c-MET (encoded by *MET*) and tyrosine kinase inhibition suppressed tumor growth by inhibiting MITF (Davis et al., 2010; Outani et al., 2014). Assessing these drugs in CreER *Ewsr1::Atf1* mouse models would determine drug response in the context of lineage-variable MITF expression.

EWSR1::ATF1 plays a significant role in regulating the chromatin environment in CCS. EWSR1::ATF1 ChIP-seq data in the TAT-Cre-induced CCS mouse tumors indicate that the fusion oncogene significantly colocalizes with H3K27ac and RNA polymerase (Ozenberger et al., 2023). EWSR1::ATF1 can promote chromatin looping to drive an epigenetic state and transcriptional program critical for CCS *in vitro* (Möller et al., 2022). EWSR1::ATF1 also physically interacts with the histone methyltransferase PRMT5 to activate its targets (Li et al., 2022a). PRMT5 inhibition is being pursued in preclinical trials for multiple malignancies and, in CCS, its chemical inhibition suppresses xenograft tumor growth (Chen et al., 2021; Li et al., 2022a).

## Common mechanisms and therapeutic opportunities

As many pediatric fusion-driven cancers contain transcription factors or chromatin regulators as a fusion partner, targeting epigenetic regulators or activated downstream pathways could be a potential therapeutic avenue.

### Epigenetic dysregulation

**Fig. 2.**
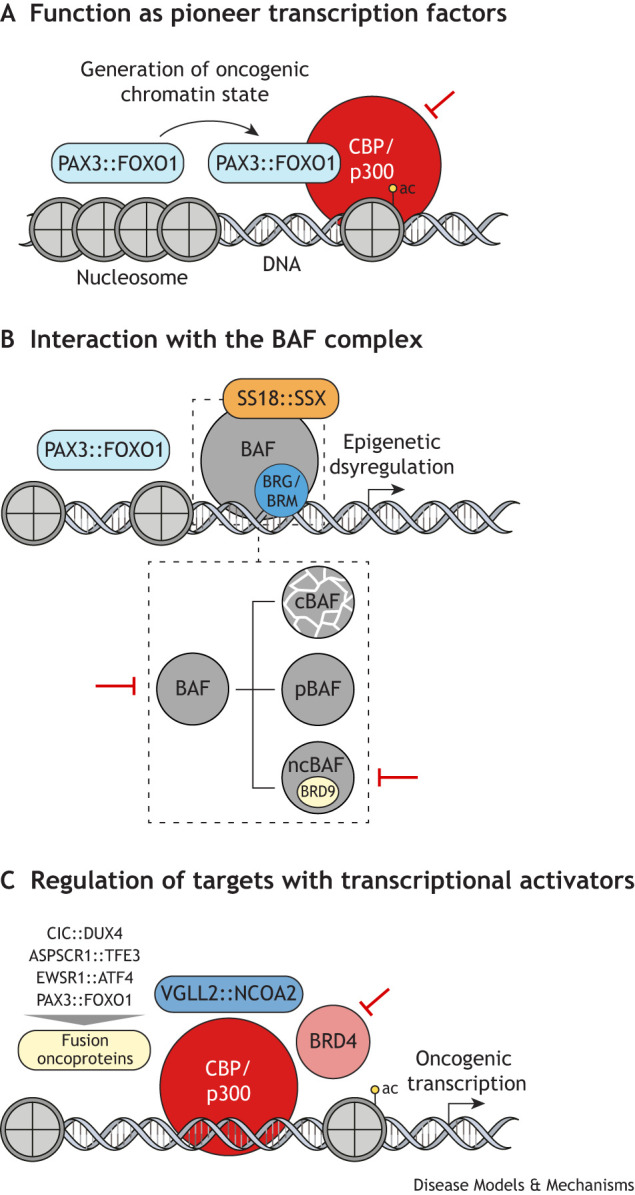
**Epigenetic regulators important across fusion oncogene-driven pediatric soft tissue sarcomas.** (A) PAX3::FOXO1 binds to inaccessible chromatin and can induce accessibility to set an oncogenic chromatin state. Transcriptional activation complexes, such as those including CBP or p300, are then recruited to promote oncogenic transcription. ac, acetylation of histone 3 at lysine 27 (H3K27ac). (B) PAX3::FOXO1 interacts with the BAF complex to regulate target genes. BRG and BRM are mutually exclusive BAF catalytic subunits. The lower dotted box shows the three main forms of the BAF complex: canonical BAF (cBAF), polybromo BAF (pBAF) and non-canonical BAF (ncBAF). SS18::SSX incorporates into the BAF complex, leading to cBAF degradation in synovial sarcoma and alteration of the relative enrichment of the BAF subcomplexes. (C) In infantile rhabdomyosarcoma, the VGLL2::NCOA2 fusion has protein domains that can interact with CBP and/or p300. The other fusion oncoproteins can bind to DNA and require CBP/p300, BRD4 and proper histone acetylation (H3K27ac) to activate their oncogenic targets. The red inhibitory signs represent targetable complexes that have been tested in preclinical studies.

The BAF chromatin remodeling and CBP/p300 transcriptional activator complexes are two epigenetic regulators with potential oncogenic roles across many pediatric fusion-driven soft tissue sarcomas (Fig. 2). The BAF complex is critical for gene regulation and is the most mutated epigenetic regulator in cancer (Kadoch et al., 2013). In FP-RMS, BAF complex protein degradation induces myogenic differentiation *in vitro* and suppresses xenograft tumor growth (Laubscher et al., 2021; Bharathy et al., 2022). Additionally, SS18::SSX causes destabilization of the cBAF subcomplex, allowing targeting of the other BAF subcomplexes through inhibition of BRD9 (Brien et al., 2018; McBride et al., 2020). However, clinical trials focused on the BAF complex, including BRD9, have faced toxicity concerns in dose-escalation studies (NCT04965753). Highlighting these potential concerns, genomic knockout of BRD9 in mice inhibited leukemia growth *in vivo* but impaired B cell development (Xiao et al., 2023). These findings highlight the need to find tumor-specific epigenetic dependencies and minimize off-target side effects of treatment.

CBP and p300 acetylate histones to promote gene expression by recruiting epigenetic readers (i.e. BRD4) and RNA polymerase II. These regulatory interactions may be especially vulnerable in pediatric cancers to activate oncogenic pathways. In preclinical studies, inhibition of BRD4 delayed FP-RMS xenograft tumor growth (Gryder et al., 2017). VGLL2::NCOA2 could directly interact with CBP and/or p300, as it retains an interacting domain from endogenous NCOA2 (Deguchi et al., 2003; Alaggio et al., 2016). This interaction could be critical in promoting oncogenic transcription as VGLL2::NCOA2 expression in mouse myoblasts increased histone acetylation at a target gene, *Arf6* (Watson et al., 2023). Moreover, inhibition of CBP and p300 suppressed CIC::DUX4-driven transcription and xenograft tumor growth (Bosnakovski et al., 2021). Both ASPSCR1::TFE3 and EWSR1::ATF1 colocalize on DNA with acetylated H3K27, suggesting that these fusions could also be susceptible to CBP or p300 inhibition (Tanaka et al., 2023; Ozenberger et al., 2023). Another strategy to block oncogenic transcription is arylstibonic acid, which inhibits the activity of bZIP transcription factors, such as EWSR1::ATF1, and inhibited CCS xenograft growth (Zhao et al., 2012).

HDAC inhibition leads to a spreading of histone acetylation across the genome and destabilization of the chromatin landscape (Gryder et al., 2019). This could be especially valuable in pediatric soft tissue sarcomas that have a distinct chromatin state. As previously discussed, altering histone acetylation with HDAC inhibitors in FP-RMS has shown some preclinical promise that still requires further evaluation (Pacenta et al., 2021; Lanzi and Cassinelli, 2022; Selim et al., 2023). *In vitro*, HDAC inhibitor treatment did not impact BRD4 phase condensates, suggesting that cancer cells remain primed to reactivate oncogenic transcriptional programs (Gryder et al., 2019). HDAC inhibitors have lacked broad success as monotherapies in fusion-driven sarcomas and could instead be used in combination therapies (Lanzi and Cassinelli, 2022; Selim et al., 2023). In SS, treating the conditional TAT-Cre *SS18::SSX2* mouse tumor model that genetically lacks *Pten* with the HDAC inhibitor quisinostat induces cell death and decreases tumor burden, suggesting potential for a combinatorial approach (Laporte et al., 2017a; Laporte et al., 2017b).

### Signaling pathways and kinases

As pediatric tumors have characteristics of aberrant development and hijack developmental pathways, their distinct reactivation in tumors could make them therapeutic targets. FP-RMS and infantile RMS fusion oncogenes both initiate molecular programs that mimic arrested skeletal muscle development. scRNA-seq from mouse xenografts identified a subpopulation of differentiated cells in tumors that were less proliferative and correlated to better outcomes (Danielli et al., 2023a). Targeting TEAD1 for myogenic differentiation therapy may be especially valuable as it interacts with VGLL2 and is an effector of the Hippo pathway, a known modifier of Pax3::Foxo1 RMS presentation in the mouse model (Maeda et al., 2002; Mesrouze et al., 2020; Oristian et al., 2018) Therefore, targeting myogenic pathways to promote RMS cell differentiation would have therapeutic benefits.

FGFR signaling is important for skeletal muscle development, and high expression of FGFR4 correlates with reduced overall survival in patients with FP-RMS (Vi et al., 2009; Ornitz and Marie, 2015). The success of preclinical pharmacological inhibition of FGFR signaling in RMS has varied by approach and is likely due to the lack of specificity of tested chemical inhibitors. A promising approach used a FGFR4-targeting chimeric antigen receptor (CAR) and found that this strategy inhibited tumor growth in metastatic and orthotopic mouse xenograft models (Wu et al., 2023; Tian et al., 2023; Milton et al., 2022). The FGFR signaling cascade is also activated in SS and its repression downregulates the MAPK pathway and *ETV4* expression (DeSalvo et al., 2021). The same downstream factors are important in URCS, as ETV4 is a primary target of CIC::DUX4 and MAPK signaling is repressed by the fusion oncoprotein (Hendrickson et al., 2024; Lin et al., 2020). Therefore, FGFR inhibition could affect multiple oncogenic pathways across these cancers, and therapeutic approaches such as CAR T-cell treatments or FGFR4 genetic cooperation could be tested in xenograft and animal models.

Finally, Wnt/β-catenin signaling is activated in SS, CIC::DUX4 and ASPS models (Barham et al., 2013; Barrott et al., 2015; Hendrickson et al., 2024). Understanding the role of Wnt/β-catenin signaling in immune evasion for ASPS will be beneficial because, even though immune therapies have shown promise, patient response rates still need improvement (Fujiwara et al., 2023). Therefore, targeting Wnt/β-catenin signaling could inhibit intrinsic tumor mechanisms and immune evasion and has promise as a combinatorial approach with immunotherapies. Recent work developed an electroporation model that could recapitulate the human diseases by delivering fusion oncogenes for FP-RMS, SS ASPS and others into mouse thigh muscles (Imle et al., 2024 preprint). The rapid generation of immunocompetent models allows for evaluation of immunotherapies and provides an exciting platform for comparative analysis.

## Concluding remarks

Pediatric fusion-driven soft tissue sarcomas remain a group of cancers with a critical unmet need for targeted therapeutics. Despite the general improvements in overall survival in pediatric cancers through the decades, soft tissue sarcomas are less responsive to standard-of-care treatments. Moreover, a deeper biological understanding of these cancers is needed to inform personalized medicine approaches and identify new therapeutic agents or repurpose existing ones. Modeling of these chimeric oncogenes in animal systems demonstrates that a specific cellular context is required for tumorigenesis. Comparative analyses across a variety of different cellular contexts will identify targetable cooperating factors and pathways that are critical for tumorigenesis. Furthermore, animal models of these diseases can preclinically evaluate agents to inform mechanisms, treatment options and pathways for resistance in a complex *in vivo* environment. However, given the broad essentiality of these epigenetic regulators and the importance of these developmental pathways, there must be a focus on tumor-specific components and interactions to limit off-target consequences. This is especially critical in children. Animal modeling is vital to fundamentally understand pediatric cancer biology in a dynamic developmental environment and to determine potential off-target toxicities and side effects. Comparing and reviewing work across these models will continue to provide valuable insights and highlight shared therapeutic opportunities to improve the outcomes for children with cancer.
